# Metabolic myopathy presenting with polyarteritis nodosa: a case report

**DOI:** 10.1186/1752-1947-5-262

**Published:** 2011-06-30

**Authors:** Sahana Vishwanath, Mishal Abdullah, Amro Elbalkhi, Julian L Ambrus

**Affiliations:** 1 Department of Medicine, SUNY at Buffalo School of Medicine, 100 High Street, Buffalo, NY 14203, USA

## Abstract

**Introduction:**

To the best of our knowledge, we describe for the first time a patient in whom an unusual metabolic myopathy was identified after failure to respond to curative therapy for a systemic vasculitis, polyarteritis nodosa. We hope this report will heighten awareness of common metabolic myopathies that may present later in life. It also speculates on the potential relationship between metabolic myopathy and systemic vasculitis.

**Case presentation:**

A 78-year-old African-American woman with a two-year history of progressive fatigue and exercise intolerance presented to our facility with new skin lesions and profound muscle weakness. Skin and muscle biopsies demonstrated a medium-sized artery vasculitis consistent with polyarteritis nodosa. Biochemical studies of the muscle revealed diminished cytochrome C oxidase activity (0.78 μmol/minute/g tissue; normal range 1.03 to 3.83 μmol/minute/g tissue), elevated acid maltase activity (23.39 μmol/minute/g tissue; normal range 1.74 to 9.98 μmol/minute/g tissue) and elevated neutral maltase activity (35.89 μmol/minute/g tissue; normal range 4.35 to 16.03 μmol/minute/g tissue). Treatment for polyarteritis nodosa with prednisone and cyclophosphamide resulted in minimal symptomatic improvement. Additional management with a diet low in complex carbohydrates and ubiquinone, creatine, carnitine, folic acid, α-lipoic acid and ribose resulted in dramatic clinical improvement.

**Conclusions:**

Our patient's initial symptoms of fatigue, exercise intolerance and progressive weakness were likely related to her complex metabolic myopathy involving both the mitochondrial respiratory chain and glycogen storage pathways. Management of our patient required treatment of both the polyarteritis nodosa as well as metabolic myopathy. Metabolic myopathies are common and should be considered in any patient with exercise intolerance. Metabolic myopathies may complicate the management of various disease states.

## Introduction

Metabolic myopathies are common disorders that are however rarely recognized in adults. They include various mitochondrial myopathies, glycogen storage diseases and disorders of purine metabolism [1,2]. Common presentations in adults may include merely exercise intolerance and muscle weakness with or without pain [3]. Patients with metabolic myopathies clear infections slowly and therefore may be more susceptible to complications of chronic infections.

Polyarteritis nodosa (PAN) is a systemic vasculitis involving medium-sized muscular arteries that has been associated with various chronic infections including hepatitis B, hepatitis C and parvovirus [4,5]. To the best of our knowledge no previous case reports or studies have examined an association between a metabolic myopathy and polyarteritis nodosa.

## Case presentation

A 78-year-old African American woman presented to our facility with a two-year history of progressively worsening fatigue and exercise intolerance. She lived alone and had been independent in her activities of daily living except for two occasions, six months and three months prior to her admission to Buffalo General Hospital, NY, USA, when she was admitted to the hospital for viral syndromes with associated muscle weakness that resolved in five to seven days. She was discharged with a diagnosis of viral syndrome and dehydration. In the three months prior to her admission to Buffalo General Hospital, she had noted progressively worsening muscle weakness and pain, increasing to the point that she was confined to a wheelchair. She had significant abdominal pain and intermittent diarrhea. Her medical history was also notable for hypothyroidism, for which she had been treated with levothyroxine replacement for 35 years, and hypertension. Her medications at the time of admission were levothyroxine 125 μg daily, atenolol 50 mg daily, aspirin 81 mg daily, calcium 500 mg daily, omeprazole 20 mg daily and a multivitamin. Her physical examination on admission was notable only for diminished muscle strength in the proximal muscles of the lower compared to the upper extremities. There was no known family history of muscle problems. Notable laboratory study results included: white blood cell count (WBC) = 31.6 × 10^9 ^cells/L, hemoglobin (HGB) = 7.7 g/dL, platelets = 464 × 10^9 ^cells/L, aspartate aminotransferase (AST) = 201 U/L, alanine aminotransferase (ALT) = 206 U/L, lactate dehydrogenase (LDH) = 273 U/L, creatine kinase (CPK) = 14 U/L, erythrocyte sedimentation rate (ESR) >150 mm/hour, C-reactive protein (CRP) = 182 mg/L, ferritin = 10,411 ng/mL, Urinalysis including microscopy was normal, thyroid stimulating hormone (TSH) = 4.27 ulU/mL, free thyroxine (T4) = 1.19 ng/dL, positive for cytoplasmic anti-neutrophil cytoplasmic antigen (C-ANCA) (>1:512), negative for perinuclear ANCA (p-ANCA), a negative hepatitis profile, positive for parvovirus IgG (3.9 index; normal: <0.9) and negative for IgM. A computerized tomography (CT) scan of the abdomen showed thickening of the colon consistent with ischemia and muscle biopsy showed vasculitis involving muscular arteries and arterioles consistent with polyarteritis nodosa. Treatment was initiated with prednisone 60 mg daily and cyclophosphamide 150 mg daily. After two weeks of therapy, minimal clinical improvement was noted, although her inflammatory parameters had decreased (WBC = 3.8 × 10^9 ^cells/L, HGB = 10.3 g/dL, platelets = 262 × 10^9 ^cells/L, ESR = 50 mm/hour, CRP = 22 mg/L, AST = 47 U/L, Alt = 23 U/L, and ferritin = 2567 ng/mL). Biochemical studies became available that demonstrated a defect in the mitochondrial respiratory chain with a low cytochrome c oxidase level of 0.78 μmol/minute/g tissue (normal range: 1.03 to 3.83 μmol/minute/g tissue), and evidence of a lysosomal defect resulting in a secondary glycogen storage disease with elevated acid maltase level 23.39 μmol/minute/g tissue (normal range: 1.74 to 9.98 μmol/minute/g tissue) and neutral maltase level 35.89 μmol/minute/g tissue (normal range: 4.35 to 16.03 μmol/minute/g tissue). Our patient was treated with a diet free of complex carbohydrates and a compound containing ubiquinone, creatine, carnitine, folic acid, α-lipoic acid and ribose, resulting in slow clinical improvement over the next several months.

## Discussion

We present the case of a patient with a complex metabolic disorder involving defects in enzymes of the mitochondrial respiratory chain and glycogen storage pathways who developed a systemic vasculitis, resulting in a need for acute medical attention. Treatment of the vasculitis resulted in improvement in laboratory parameters but not in clinical symptoms. Symptomatic improvement occurred with additional management of the complex metabolic disease.

Several metabolic diseases are known to present in adulthood and to be common in the general population. Myoadenylate deaminase deficiency affects approximately 5% of the population, myophosphorylase deficiency, a glycogen storage disease, affects 8% of the population and various mitochondrial disorders exist in between 1:1000 to 1:10,000 of the population, depending upon various estimates [1,6-8]. Our patient had a mitochondrial respiratory chain defect along with an unusual glycogen storage defect with high levels of lysosomal acid and neutral maltase, likely resulting in reduced cytosolic levels of these enzymes. It is possible that the mitochondrial defect resulted from inadequately replaced hypothyroidism, but there is no data to support this hypothesis and our patient's thyroid studies were normal at the time of admission [9]. The manifestations of these metabolic diseases in an adult are often merely exercise intolerance and fatigue, which were getting worse in our patient over a period of two years [1]. At the same time, patients with metabolic diseases often have difficulty clearing infections, and our patient had two admissions for worsening muscle symptoms because of viral infections even before the onset of her vasculitis [10]. Interestingly, our patient did have positive IgG serology results for parvovirus, which has been associated with polyarteritis nodosa [5,11]. It is possible that difficulty with clearing parvovirus led to immune complex formation and secondary vasculitis, although there was no evidence of ongoing parvovirus infection at her time of admission to Buffalo General Hospital.

The manifestations of polyarteritis nodosa in our patient were muscle pain and weakness, abdominal pain and elevated inflammatory markers including ESR, CRP, ferritin and platelets. She had a positive C-ANCA result that is most commonly associated with Wegener granulomatosis but can certainly be seen in polyarteritis nodosa as well [12]. Our patient had a clinical picture and muscle biopsy supporting a diagnosis of polyarteritis nodosa but not Wegener granulomatosis. Interestingly, treatment for polyarteritis nodosa, high-dose steroids and cyclophosphamide, resulted in improvement in inflammatory parameters with resolution of abdominal symptoms, but not muscle weakness. Improvement in muscle weakness occurred over a period of months with management of the metabolic disease.

The management of mitochondrial dysfunction is currently under investigation, but several approaches have been shown to be fruitful. Supplementing with ubiquinone (CoQ10), which transports electrons between complex I and complex III of the mitochondrial respiratory chain, has been shown to improve mitochondrial function in several studies [13]. Creatine is utilized to generate additional ATP through the creatine phosphate shuttle [14]. Carnitine is added to enhance transport of fatty acids into the mitochondria. Folic acid is a cofactor for several mitochondrial enzymes. α-Lipoic acid is a strong antioxidant [15]. In the management of glycogen storage diseases, complex carbohydrates are avoided and simple sugars, such as ribose, are utilized to provide a more available energy source [3,16]. This strategy resulted in significant symptomatic improvement in our patient.

It has recently been noted that patients who have inflammatory muscle diseases that do not respond to immunosuppressive therapies as expected often have underlying metabolic muscle diseases. This is the first documented case of a vasculitis with incomplete clinical response to immunosuppressive therapy in which the management of a complex metabolic disorder was necessary for symptomatic relief [17,18]. This case should alert physicians to include various common adult onset metabolic disorders in the investigation of symptom complexes including fatigue and various muscle problems.

## Conclusions

To the best of our knowledge, this report describes for the first time a patient in which symptomatic improvement of a systemic vasculitis required the simultaneous management of a metabolic myopathy. Since metabolic myopathies are common, they should always be considered when inflammatory diseases are not responding to standard therapies as expected.

## Consent

Written informed consent was obtained from the patient for publication of this case report and any accompanying images. A copy of the written consent is available for review by the Editor-in-Chief of this journal.

## Competing interests

The authors declare that they have no competing interests.

## Authors' contributions

All authors participated in the care of our patient and the writing of this manuscript. All authors read and approved the final manuscript.
